# Metagenome-assembled genomes from oxygenic photogranules obtained from photobioreactors treating synthetic wastewater

**DOI:** 10.1128/mra.01310-25

**Published:** 2026-02-04

**Authors:** Oriane Della-Negra, Rémi Servien, Kim Milferstedt, Jérôme Hamelin, Christophe Klopp, Claire Hoede

**Affiliations:** 1 INRAE, University of Montpellier, LBE, Narbonne, France; 2 University of Toulouse, INRAE, UR 875 MIAT, Castanet-Tolosan, France; 3 University of Toulouse, INRAE, BioinfOmics, GenoToul Bioinformatics Facility, Castanet-Tolosan, France; 4 University of Toulouse, INRAE, BioinfOmics, Sigenae, Castanet-Tolosan, France; DOE Joint Genome Institute, Berkeley, California, USA

**Keywords:** photogranules, MAGs, wastewater treatment, nitrogen metabolism

## Abstract

Twenty-five high-quality metagenome-assembled genomes (MAGs) were recovered from photogranules to treat synthetic wastewater. They were dominated by *Leptolyngbya boryana*. Cyanobacterial MAGs encoded photosynthesis and nitrogen fixation pathways, supporting internal oxygen and nitrogen cycling. Most heterotrophic MAGs contributed to nitrogen removal, highlighting the metabolic complementarity within photogranules studied for wastewater treatment.

## ANNOUNCEMENT

Photogranules are phototrophic microbial aggregates varying from 200 µm to millimeters in size (1). They represent a candidate biomass for future wastewater treatment (2). They consist of syntrophically interacting cyanobacteria and heterotrophic bacteria, in which cyanobacteria produce oxygen while heterotrophs provide carbon dioxide (3).

Photogranules originated from a 4 L photobioreactor fed with synthetic wastewater, as previously described (4), and were grown in a sequencing batch reactor with synthetic wastewater containing acetate and ammonium. Cool white light was provided at 96 µmol m⁻² s⁻¹ of photosynthetically active radiation. After 400 days of operation, photogranules were collected, sieved to a diameter of 0.6–1 mm, and about 500 photogranules were stored at −70°C prior to DNA extraction.

High-molecular-weight DNA was extracted using the FastDNA Spin Kit for Soil (MP Biomedicals, USA), with manual cryo-grinding in liquid nitrogen replacing bead beating. DNA (average size 9.9 kb) was purified with AMPure PacBio beads (0.45× vol/vol). Metagenomic libraries were prepared using the SMRTbell preparation workflow and PacBio Revio Polymerase Kit (Pacific Biosciences, USA), without DNA shearing. Unlike the standard protocol, DNA fragments <5 kb were removed prior to library construction using diluted AMPure PacBio beads (3.1× vol/vol, 35% in TE). Sequencing was performed using PacBio Revio technology at the Gentyane INRAE platform (Clermont-Ferrand, France), yielding reads with an *N*_50_ of 10,184 bp.

Quality filtering and adapter removal were conducted using SMRT Link (v.13.1.0.221970), yielding 1,814,020 HiFi reads. Reads were processed using the metagWGS pipeline v 2.4.3 (5), and assembled with metaMDBG v1.0 (6). Contigs were filtered, annotated, taxonomically assigned, and binned with metagWGS (default parameters, *N*_50_ = 407.7 Kbp, assembly length = 350 Mbp, % reads mapped = 99.6%, and number of contigs = 5,770).

Bins were refined with binette v0.1.5 (7), dereplicated with dRep v3.0.0 (95% threshold), and quality-checked with CheckM2 v1.0.2 (8). High-quality metagenome-assembled genomes (MAGs) (>90% completion, <5% contamination) were annotated with Prokka v1.15.5 (9), classified using GTDB-Tk v2.1.1 (10), and functionally annotated using eggNOG-mapper v2.1.9 (11). Phylogenetic analysis was based on 43 universal marker genes aligned with msa R-package v1.28 (12), and a maximum-likelihood tree was inferred with phangorn v2.11.1 (13). Comparative features of MAGs are summarized in Table 1.

**TABLE 1:** Genomic features of the MAGs recovered from oxygenic photogranules annotated by Prokka v1.15.5 and metagWGS pipeline v.2.4.3 (5)

	BioSample accession no. SAMN	CheckM completeness (%)	CheckM contamination (%)	No. of contigs	Genome size (bp)	*N*_50_ (bp)	G + C%	Total CDS	Total genes	RNA genes	No. of tRNA	No. of ncRNA	No. of 16S rRNA	No. of 23S rRNA	No. of 5S rRNA	GTDB-Tk taxonomy v2.1.1
*sample1_bin_104*	50846517	100.0	0.5	1	3,373,197	3,373,197	32.0	3,014	3,050	36	35	1	2	2	2	f__UBA8524
*sample1_bin_12*	50846518	100.0	0.0	4	3,892,925	919,946	64.9	3,675	3,730	55	54	1	5	4	5	s__Hydrogenophaga sp021166755
*sample1_bin_17*	50846519	99.7	0.1	1	3,158,304	3,158,304	58.7	3,038	3,087	49	48	1	1	1	1	s__UphvI-Ar2 sp020161505
*sample1_bin_20*	50846520	91.7	3.5	5	3,705,667	1,954,446	63.3	3,501	3,560	59	58	1	3	3	3	g__Azonexus
*sample1_bin_2886*	50846521	100.0	0.8	2	4,654,488	4,614,637	68.2	4,062	4,131	69	68	1	4	4	4	s__Thauera sp016790365
*sample1_bin_2887*	50846522	100.0	0.0	4	3,402,847	3,123,638	61.8	3,244	3,292	48	47	1	1	1	1	s__Rhabdaerophilum sp017302635
*sample1_bin_2913*	50846523	99.7	0.9	6	4,565,454	993,269	60.8	4,336	4,394	58	57	1	2	2	2	g__Azonexus
*sample1_bin_33*	50846524	99.9	0.1	1	1,727,191	1,727,191	41.5	4,004	4,052	48	48	0	1	1	1	f__Nucleicultricaceae
*sample1_bin_3387*	50846525	99.4	0.6	11	3,947,745	2,266,092	62.9	1,586	1,628	42	42	0	2	2	2	s__Vitreimonas flagellata
*sample1_bin_37*	50846526	100.0	0.0	1	4,030,457	4,030,457	42.4	3,132	3,168	36	35	1	1	1	1	g__UBA4660
*sample1_bin_41*	50846527	99.8	0.6	4	7,018,121	6,309211	46.9	6,473	6,543	70	69	1	3	3	3	s__Leptolyngbya boryana
*sample1_bin_5*	50846528	99.8	0.5	3	6,874,972	6,228,686	46.9	3,715	3,754	39	38	1	2	2	2	s__Leptolyngbya boryana
*sample1_bin_56*	50846529	100.0	0.0	1	4,507,676	4,507,676	46.3	6,398	6,467	69	68	1	3	3	3	s__Phnomibacter sp020848815
*sample1_bin_63*	50846530	98.2	0	1	4,362,800	4,362,800	55.9	3,691	3,738	47	47	0	2	2	2	f__Phototrophicaceae
*sample1_bin_65*	50846531	100.0	0.2	4	4,592,462	3,912,904	65.9	4,434	4,490	56	55	1	2	2	2	g__Pseudogemmobacter
*sample1_bin_70*	50846532	99.8	0.7	1	1,799,130	1,799,130	56.2	5,346	5,426	80	79	1	3	4	8	o__UBA9219
*sample1_bin_7088*	50846533	98.3	1.0	12	6,552,131	6,427,631	53.1	1,829	1,872	43	42	1	1	1	1	g__Pirellula_B
*sample1_bin_74*	50846534	93.3	0.6	1	4,518,967	4,518,967	62.6	3,723	3,771	48	48	0	1	1	1	g__BJHL01
*sample1_bin_76*	50846535	100.0	0.1	1	4,212,359	4,212,359	63.7	4,060	4,113	53	52	1	2	2	2	s__Gemmobacter_B sp022843025
*sample1_bin_82*	50846536	100.0	0.9	22	3,586,543	278,913	69.1	3,597	3,648	51	50	1	1	1	1	g__Caulobacter
*sample1_bin_84*	50846537	97.2	0.1	9	3,093,156	644,857	39.6	3,716	3,760	44	43	1	4	4	4	s__Legionella lytica
*sample1_bin_85*	50846538	99.9	0.3	21	3,521,353	258,949	63.1	3,445	3,493	48	47	1	1	1	1	g__Hyphomonas
*sample1_bin_91*	50846539	98.9	1.2	27	3,322,966	246,897	56.5	3,251	3,295	44	43	1	1	1	1	s__Sphingorhabdus_B lacus
*sample1_bin_92*	50846540	96.7	0.5	1	1,428,886	1,428,886	52.3	1,464	1,503	39	38	1	1	1	1	o__UBA1280
*sample1_bin_99*	50846541	100.0	0.2	1	4,088,815	4,088,815	68.1	3,686	3,739	53	52	1	1	1	1	f__Steroidobacteraceae

**Fig 1:**
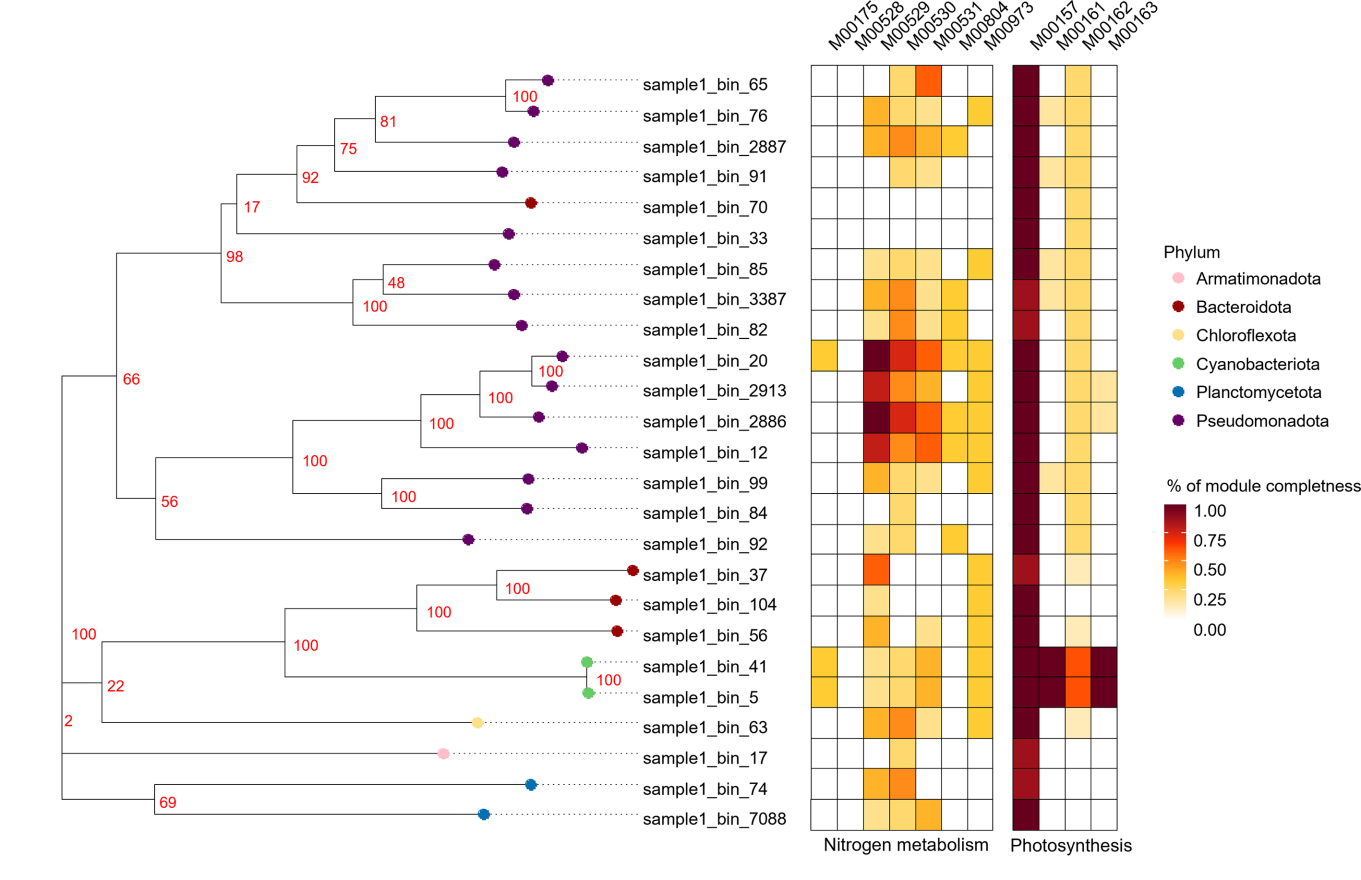
Phylogenetic tree for the 25 MAGs from the oxygenic photogranules. Heatmaps show the module completeness obtained from eggNOG-mapper annotation, associated with Nitrogen Metabolism (ko 00910) and Photosynthesis (ko 00195).

Among the 25 MAGs, 23 contained genes related to nitrogen metabolism. No KEGG nitrogen metabolism genes (map00910) were detected in Nucleicultricaceae (bin 33) or UBA9219 (bin 70), suggesting reliance on organic nitrogen sources or specialized metabolic capacities. Nucleicultricaceae, an amoebae endosymbiont, possesses a reduced genome (1.73 Mbp), suggesting metabolic dependency on a host such as *Amoebozoa* sp. detected in the metagenome (14). MAGs of Hydrogenophaga, Thauera, and Azonexus (bins 12, 20, 2,886, and 2,913; Table 1) contained near-complete denitrification modules (>80%, M00529; Fig. 1) and partial gene repertoires (33–75%) for other nitrogen pathways, highlighting their role in nitrogen removal. These organisms encode both aerobic and anaerobic metabolic genes, consistent with the oxic outer layer and anoxic core of photogranules. Cyanobacterial MAGs (bins 5 and 41), affiliated with *Leptolyngbya boryana*, contained nitrogen fixation genes (M00175) and an almost complete photosynthesis genes repertoire (Fig. 1), with no evidence of cyanobacterial toxin production. Taken together, the 25 MAGs represented 82% of the total microbial reads and 82% of bacterial abundance based on 16S rRNA gene sequencing (SRA: SRR35213077 and SRR35213076).

## Data Availability

All sequencing data are available in the Sequence Read Archive (SRA) NCBI database under BioProject accession PRJNA1297605. The raw metagenomic reads are available under BioSample accession SAMN50229464 and SRA SRR36402021. The assembled metagenomic reads are deposited under BioSample accession SAMN50229464, and the metagenome-assembled genomes (MAGs) are available under BioSample accessions SAMN50846517–SAMN50846541. Fasta files for assembled metagenome and MAGs are also available in the Zenodo repository 10.5281/zenodo.17911224. The 16S rRNA gene sequences are available at the SRA under accession numbers SRR35213077 and SRR35213076.
